# HMTI-0197. Whole blood transcriptome analysis in migraine with aura patients: a case control study

**DOI:** 10.1186/1129-2377-15-S1-B24

**Published:** 2014-09-18

**Authors:** A Oterino, M Toriello, A Esteve-Codina, S Heath, J Castillo, J Fernandez, E Pons, S Gutierrez, E Palacio, VG Quintanilla

**Affiliations:** 1 Neurology, Hospital Universitario Marqués de Valdecilla, Santander, Spain; 2 Functional Bioinformatics Team, Centre Nacional d’Anàlisi Genòmica, Barcelona, Spain; 3 Servicio Cántabro de Salud, Camargo Costa Health Center, Camargo, Spain

## Antecedents

There has been only one report analyzing whole blood transcriptome (WBT) in menstrual migraine.

## Objective

To investigate interictal WBT in patients suffering from migraine with aura (MWA).

## Methods

We performed 52 RNA-seq experiments with 26 controls (20 females) and 26 interictally-drawn samples (20 females) from sex- and age-adjusted patients suffering from MWA using Illumina Hi-seq high-throughput sequencing machine. To detect differentially expressed (DE) genes between affected and unaffected samples we used the edgeR (robust dispersion estimation) software correcting by sex.

## Results

We obtained more than 100 million short reads per sample. We identified 93 up-regulated genes in unffecteds, and 7 up-regulated genes in affecteds (FDR< 5%). Interestingly, among those DE genes we found some related to endothelial function (HBG1, HBG2, and ADIPOR1), and other related to solute carriers, energetic mechanisms, and apoptosis were also found up-regulared in controls; overexpression of genes involved in inflamation were found in affecteds. We also performed a gene ontology enrichment for the differentially expressed genes (DAVID database) and found eritrocyte and myeloid differentiation processes to be significant (Bonferroni <0.1). Other gene ontologies, like apoptosis or positive regulation of synaptic transmission, showed uncorrected significant P values (<0.05).

## Conclusion

Our preliminary results after analyzing WBT found 100 differentially expressed genes in MWA. The most significantly DE genes are involved in endothelial homeostasis. These results open new targets for understanding migraine pathophysiology.

Funded by grants from ISCIII FISS PI11/1232 and IFIMAV.

No conflict of interest.

